# Community Health Benefits Through a Student-Run Nonprofit Pediatric Wellness Clinic

**DOI:** 10.7759/cureus.60085

**Published:** 2024-05-11

**Authors:** Margaret L Munz, Owen L Young, Alexis M Stoner, David Redden

**Affiliations:** 1 Surgery, Edward Via College of Osteopathic Medicine, Spartanburg, USA; 2 Family Medicine, Edward Via College of Osteopathic Medicine, Spartanburg, USA; 3 Preventive Medicine and Public Health, Edward Via College of Osteopathic Medicine, Spartanburg, USA; 4 Biomedical Affairs and Research, Edward Via College of Osteopathic Medicine, Auburn, USA

**Keywords:** preventive medicine, osteopathic education, underserved populations, pediatric screening, pediatrics primary care

## Abstract

Purpose

Community screening programs have been in effect since they were utilized in the 19th century at county fairs. A free pediatric health screening program was created by an osteopathic medical school in South Carolina in collaboration with a pediatric dental outreach organization to engage the local underserved community and train community-minded medical professionals. This study sought to demonstrate the efficacy and need for a student-run monthly pediatric health screening program in an underserved pediatric demographic.

Methods

A retrospective study of preexisting de-identified data obtained from a student-run health screening program was analyzed to determine the efficacy of the screening program in detecting chronic health risk factors in children in an underserved population. Patients were recruited through a partnership with a free dental clinic for underserved and uninsured children. Patients who attended the clinic were offered the opportunity to have a free, comprehensive health assessment following their dental visit. The function of this program was unique in that uninsured, underserved patients were provided free dental care and a free health assessment. Pediatric patients were screened for basic health information such as weight, height, BMI, vision, cardiovascular health, hypertension, asthma (reported via questionnaire by either the parent or child when applicable), nutrition, and lead poisoning (via questionnaire). The program also offered families additional support by connecting them to local resources and answering any questions they had about their children’s health. Data from 14 health screening events was collected for quality improvement and efficacy monitoring. Descriptive analyses were performed.

Results and analysis

The health screening program assessed 124 children between October 2021 and March 2023 over 14 health screening events. The patients ranged from one year old to 26 years old, with a mean age of 9.65 years. Patients were predominantly Hispanic (79.67%). About one-third (27.64%) of children who were screened had positive findings associated with increased risk for chronic disease. Nearly half (43.90%) of families that were screened requested further information on ways to obtain health insurance and regular primary care services (utilized Access Health). Of the one-third of children with positive risk factors, 12.20% reported positive findings associated with asthma. Of the patients with positive risk factors, 8.94% had vision abnormalities, most of whom had not been seen by an ophthalmologist. This preliminary analysis will be followed by a secondary analysis that further investigates patient demographics (primarily Hispanic) as well as age distribution across various risk factors.

Conclusion

This pediatric health screening program has demonstrated a basic level of efficacy by successfully identifying increased risk for chronic disease in the underserved pediatric population. The need for these screening events was highlighted by the identification of untreated positive findings.

## Introduction

According to recent data, approximately 40% of children in the United States are affected by at least one chronic disease [1]. The consequences of uncontrolled childhood health conditions lead to progressive chronic disease in adulthood that increases the burden on the health system. In the United States, chronic diseases are the main cause of poor health and death [2]. Treatment of chronic diseases has been estimated to directly cost the healthcare system more than $1 trillion [3]. One of the major drivers of this cost is the lack of use of preventive care services, largely due to the inaction of medical providers [3]. Some data estimate that 75% of healthcare expenditure is spent to treat health conditions that are preventable, while only 3% of expenditure is spent trying to prevent disease from occurring in the first place [4].

Addressing risk factors with a preventive approach instead of a retroactive approach is crucial to preventing adverse outcomes in adulthood. Identifying children with asthma and providing their families with education on this risk can reduce pulmonary illness in adulthood [5]. A study published in the American Heart Association journal on hypertension found that elevated blood pressure in childhood is significantly associated with detrimental cardiovascular outcomes in adulthood [6]. However, children continue to face barriers to care and primary care physicians who can catch and treat these chronic illnesses early. In particular, racial and ethnic minority children experience greater difficulties in accessing care [7].

It is known that children who are covered by insurance are more likely to utilize healthcare services, including preventive services, as compared to those who are uninsured [8]. As such, multiple attempts to reduce the prevalence of children without primary care have been made through broadening insurance coverage for children. Starting with Medicaid in 1965, children of dependent families falling below the poverty line have been covered by government insurance. Then, in 1997, the Children’s Health Insurance Program (CHIP) was created to expand coverage and insure children of families that made too much to qualify for Medicaid. Today, coverage continues to expand but faces challenges. The Families First Coronavirus Response Act (FFCRA), enacted in response to the coronavirus pandemic, expanded enrollment in Medicaid and likely led to an increase in coverage in 2020 following a period of coverage loss from 2016 to 2020, as the Kaiser Family Foundation (KFF) estimates 7.3 million children gained coverage from February 2020 to March 2023 [9,10]. However, as part of the Consolidated Appropriations Act, Medicaid enrollment provisions will be decoupled from the FFCRA, and many people will be unenrolled from Medicaid, reversing the gains made [10]. States have been given the option to renew Medicaid eligibility; however, 10 states have not, including South Carolina [11].

Health community screening programs have been effective since they were utilized in the 19th century at county fairs and serve as a way to screen for risk factors for chronic illness and connect people to healthcare [12]. The use of community health screening events conducted by student health professionals has been shown in other studies to both increase community engagement and have a positive benefit for medical education [13]. There is evidence that community engagement can decrease the risk of developing chronic disease, particularly for minority groups [14]. Studies have further shown that communities lacking resources have poorer health outcomes [15]. Therefore, health screening fairs provided to the community at no cost serve as key mediators to improve health outcomes, especially for poorly resourced communities. The benefit of utilizing health fairs has been found to be appreciated by participants, as noted by one study that followed up with individuals who participated in a cancer screening event [14]. The researchers found that most participants in these health fairs believed they benefited from the event and were motivated to take action, further highlighting how health fairs can effectively educate the community they engage [14]. This study sought to demonstrate the efficacy and need for a student-run monthly pediatric health screening program in an underserved pediatric demographic.

## Materials and methods

A retrospective study of data obtained from an osteopathic medical student-run health screening program was analyzed to determine the efficacy of the screening program in detecting chronic health risk factors in children in an underserved population. After review by the Edward Via College of Osteopathic Medicine Institutional Review Board, this project was deemed exempt, and as such, informed consent was not required. Patients were recruited at 14 screening clinics from October 2021 to March 2022 through a partnership with a nonprofit dental clinic that provides free dental care at a local community college in upstate South Carolina. The dental clinic screened for dental abnormalities at local schools in grades K-12 and then referred patients without insurance who produced any positive dental abnormalities to their established clinic for further workups.

A volunteer medical outreach program at a local osteopathic medical school created monthly, ongoing health screening events that took place during dental clinic hours. Patients who attended the clinic were then offered the opportunity to have a free health assessment following their dental visit. The function of this program was unique in that, through a partnership with a dental clinic, uninsured, underserved patients were provided free dental care and a free health assessment. Pediatric patients were screened for basic health information such as weight, height, BMI, vision, cardiovascular health, hypertension, asthma, nutrition, and lead poisoning. Asthma was assessed using the Children’s Hospital of Pittsburgh Asthma Screening Questionnaire, and lead poisoning risk was assessed using the South Carolina Department of Health and Environmental Control Lead Exposure Questionnaire [16,17]. The program also offered families additional support by connecting them to local resources and answering any questions they had about their children’s health. Additional educational components addressed lead exposure risks, nutrition, and mental health. After the screening was completed, patients and their families were put in contact with healthcare resources to help connect them with primary care providers.

Access Health, an organization started in 2010, guides families without health insurance to physicians and other health or social services. A major function of the monthly screening clinics was to connect families in need of health care to Access Health [8]. Data from 14 health screening events was collected for quality improvement and efficacy monitoring. Descriptive analyses were performed. A child had a positive screen if they produced one or more findings associated with an increased risk of chronic disease. The individual demographics were analyzed, such as age and ethnicity, as well as pertinent positive risk factors and a total number of risk factors. The ethnicity of the participants was recorded from self-reported data by the patients themselves or family members. The ethnicity and demographic data were collected to better understand the patient population and assess the clinic’s efficacy in an underserved population. Pertinent positive risk factors were defined as clinical information gathered at the screening that signifies a significant risk to the child’s current or future health.

## Results

**Figure 1:**
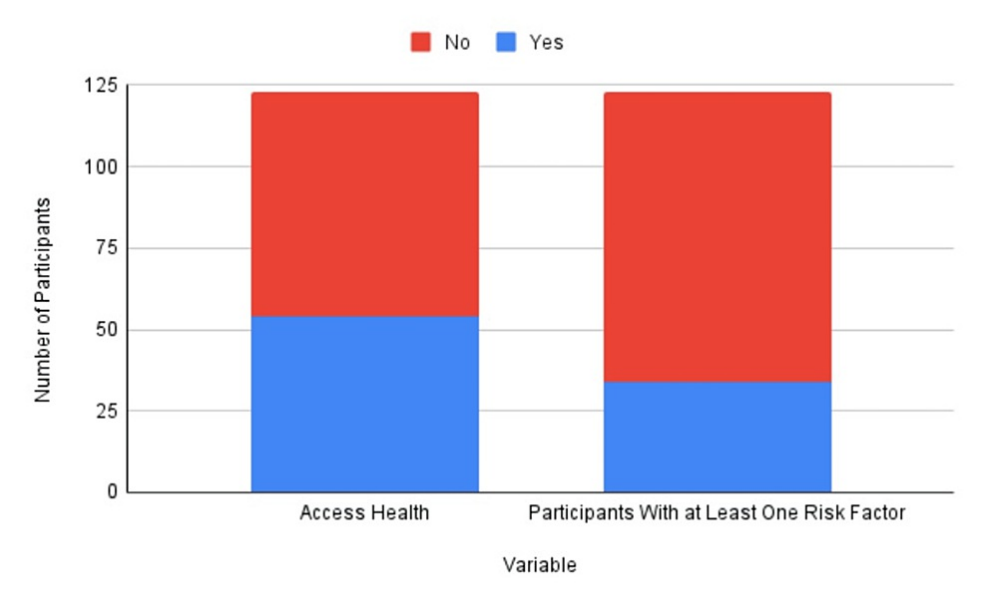
Presence of risk factors and utilization of health resources

**Figure 2:**
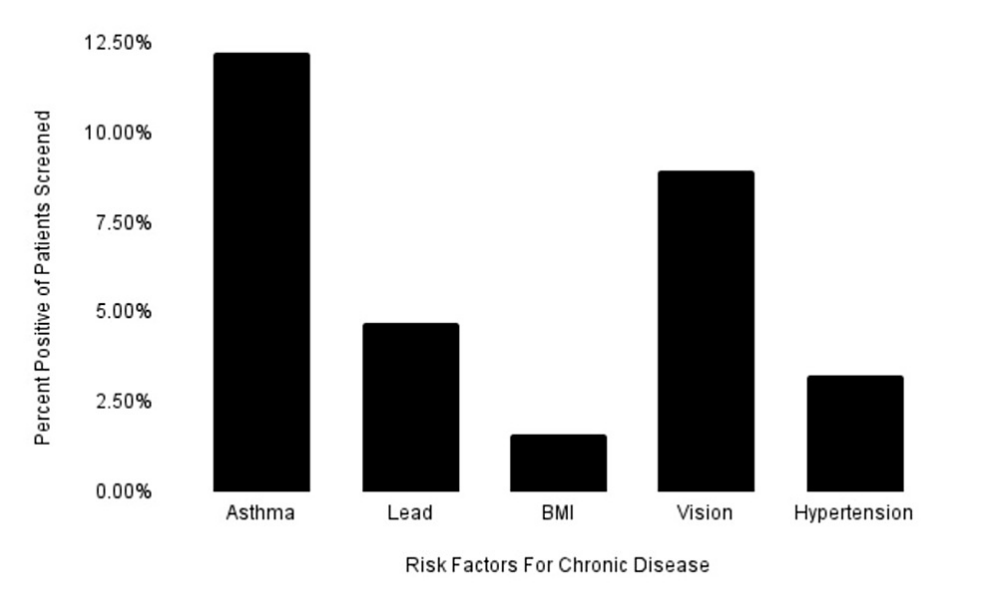
Prevalence of pertinent risk factors for chronic disease in the health screening patient population, including asthma, lead poisoning, BMI, vision abnormalities, and hypertension

The health screening program assessed 124 children between October 2021 and March 2023 over 14 health screening events. The patients ranged from one year old to 26 years old, with a mean of 9.65 years old (Table 1). Patients were predominantly Hispanic (79.67%) (Table 2). The second largest demographic was non-Hispanic (13.10%), while 7.32% were unrecorded. Some patients were either uncomfortable disclosing their ethnicity or were unable to do so due to time constraints (Table 2). About one-third (27.64%) of children who were screened had positive findings associated with increased risk for chronic disease (Figure 1). Nearly half (43.90%) of families that were screened requested further information on ways to obtain health insurance and regular primary care services (utilized Access Health) (Figure 1). Of the one-third of children with positive risk factors, 12.20% reported positive findings associated with asthma (Figure 2). Of the patients with positive risk factors, 8.94% had vision abnormalities, most of whom had not been seen by an ophthalmologist (Figure 2).

**Table 1:** Age distribution of the patient population N = total number of individuals

Variable	N	Mean	Standard deviation	Minimum	Maximum
Age	121	9.65	4.26	1	26

**Table 2:** Ethnicity demographic data of participants

Ethnicity	Number of individuals	Fraction of the population
Hispanic	98	79.67%
Non-Hispanic	16	13.10%
Not recorded	9	7.32%

## Discussion

Within the constraints of this study, our results indicate that a nonprofit monthly pediatric health screening event was effective in identifying positive risk factors for chronic disease in children that could otherwise have gone unnoticed. While the majority of patients did not have positive risk factors, we were able to demonstrate that the health screening events identified positive risk factors, particularly in a minority group.

Our research further supports the data that asthma is one of the most prevalent chronic illnesses in the US and follows trends along ethnic lines [4]. The reasons for this are multifactorial, but one trend worth noting is that the prevalence of asthma is inversely related to income (higher-income families have lower asthma rates) and unrelated to ethnicity [18].

The number of families requesting more information about Access Health supports previous data that children in South Carolina are in need of a healthcare provider and are likely facing barriers to care due to insurance [19]. Just as important as identifying health risk factors is having a follow-up with a physician who can manage the identified health issues. The role of Access Health is one that needs to be modeled at other health screening programs in order to have a lasting effect on the population being served.

In addition to serving underserved community members, these monthly screening events have the added benefit of allowing osteopathic medical students to volunteer in the clinical setting. These early clinical exposures not only help improve clinical skills but also help improve students’ communication with patients and encourage an attitude of philanthropy. This affirms the values instilled in osteopathic medical students as outlined by the osteopathic oath [20]. Osteopathic medical training encourages a focus on the whole person, including a focus on preventive care and training toward primary care [21]. Osteopathic medical schools comprise eight of the top 10 medical schools in the United States producing graduates that go into primary care and three of the top 10 schools that produce graduates serving in underserved areas [22]. Therefore, exposing students to the health needs of an underserved community through extracurricular activities such as this health screening event provides an avenue for training students to become effective, community-minded primary care physicians and fulfills the primary goal of osteopathic medical training.

Limitations in this study include a lack of follow-up with families using Access Health due to patient privacy restrictions. Data was collected for quality assessment and basic efficacy; therefore, procedures to collect information were not thoroughly controlled among data gatherers. Future endeavors should include ways to follow up with patients outside of the screening clinic that follow patient privacy rules. As well, data collection should be standardized among students gathering information from patients.

## Conclusions

This pediatric health screening program has demonstrated a basic level of efficacy by successfully identifying increased risk for chronic disease in the underserved pediatric population. Additionally, the screening events provided patient education and an opportunity to establish a healthcare provider for the uninsured while allowing osteopathic medical students to volunteer and practice clinical skills and communication. The need for these screening events was highlighted by the identification of positive health risk factors for chronic disease, including increased BMI, hypertension, and diminished vision, among others. Future studies to identify if patients can establish care through Access Health and maintain continuity of care would be valuable. This would support the potential expansion of the monthly screening events and their critical role in bridging the gaps between underserved community members and vulnerable populations, such as the pediatric population, and healthcare access.
